# Looking to Learn Better - Training of Perception-Specific Focus of Attention Influences Quiet Eye Duration but Not Throwing Accuracy in Darts

**DOI:** 10.3389/fspor.2020.00079

**Published:** 2020-07-01

**Authors:** Judith Neugebauer, Joseph Baker, Jörg Schorer

**Affiliations:** ^1^Institute of Sport Science, University of Oldenburg, Oldenburg, Germany; ^2^School of Kinesiology and Health Science, York University, Toronto, ON, Canada

**Keywords:** quiet eye, focus of attention, motor learning, vision, instructions

## Abstract

Investigations of the association of focus of attention and quiet eye duration have shown mixed results. It is possible that when focusing on visuo-motor learning a more sensitive category system of instructions might be useful. The aim of this study was to investigate the interaction of focus of attention and quiet eye duration in darts. In addition to locus-directed foci (external, internal), perception-directed (visual, kinesthetic) foci of attention were considered. Participants were divided into four groups and had to perform a pre- and post-test with a 1-week training intervention in between. Throwing accuracy (TA) and quiet eye duration (QED) were measured using the SMI eye tracking glasses. An analysis of covariance (2x2) showed no significant group differences or interactions for TA. For QED, an analysis of variance (2x2x2) showed quiet eye duration was increased with the intervention but there were significant differences between the tests. A significant interaction of test and perception-directed focus was observed. Visually instructed groups increased QED whereas the kinesthetic group decreased the QED, suggesting perceptual and motor learning may be asynchronous. One possible explanation for the trends might be the common-coding theory of perception and action.

## Introduction

Sport research has shown that perceptual and motor performance are strongly linked (Janelle et al., 2000; Martell and Vickers, 2004; Vickers and Williams, 2007; Lohse et al., 2010; Wulf, 2013). For example, the “quiet eye” is a perceptual phenomenon that considers the influence of final fixation duration on motor learning and performance processes (for an overview, Vickers, 2007; Lebeau et al., 2016). Similarly, a performer's “focus of attention” in the context of motor learning and motor performance has received considerable attention in the last two decades (for a review, Wulf, 2013). In the past few years, several studies have considered the relationship between quiet eye and focus of attention, showing mixed results (Moore et al., 2012; Klostermann et al., 2014; Rienhoff et al., 2015). The main objective of the present study was to gain deeper insight into the relationship between focus of attention and quiet eye, particularly the interdependency of motor and perceptual learning, by considering the influence of different instructions on quiet eye duration and motor performance in a learning task.

Based on the ground-breaking work by Wulf et al. (1998), a range of researchers have considered the influence of external (i.e., attention to one's movement effect) and internal (attention to one's own movement) foci of attention in sport situations (Perkins-Ceccato et al., 2003; Wulf, 2013). Most of these studies have shown a benefit for an external focus of attention in the context of motor learning (Carpenter et al., 2013; Wulf, 2013), although some studies have shown inconsistent results (Rienhoff et al., 2015).

A few studies have considered the influence of focus of attention instructions in aiming tasks (Zachry et al., 2005; Castaneda and Gray, 2007; Wulf and Su, 2007; Lohse et al., 2010; Schorer et al., 2012; Querfurth et al., 2016). Schorer et al. (2012), for example, compared the influence of different instructions on dart throwing performance of experts and novices, finding mixed results. In doing so, they used two internal and one external focus of attention conditions. Castaneda and Gray (2007) compared skill/external, skill/internal, environmental/irrelevant and environmental/external instructions in less-skilled and highly skilled baseball players. For highly-skilled athletes, they noted a benefit for an environmental/external instruction; for the less-skilled athletes they found best performances in the two skill conditions. Wulf and Su (2007) investigated the influence of external and internal instructions on golf shooting accuracy in novices in a learning task. The novices were divided into three groups (internal, external, control) and performed 60 practice and 10 set trails. Results indicated a benefit of an external focus of attention. Finally, Lohse et al. (2010) investigated participants in a dart throwing task and compared internal and external instructions. Equally, benefits for an external focus on dart throwing performance were identified. As noted earlier, the results of these studies have been mixed, which may have been due to the inconsistency in the instructions used (see Table 1).

**Table 1:** Varying used focus of attention instructions in aiming tasks.

**Study**	**External instruction**	**Internal instruction**	**Supplementary instruction**
Castaneda and Gray (2007) Participants: less-skilled vs. highly skilled	Skill/external: movement of the bat (kinesthetic) Environmental/external: the ball leaving the bat (kinesthetic)	Skill/internal: movement of the hand (kinesthetic)	Environmental/irrelevant: auditory tones
Wulf and Su (2007) Participants: Novices	Pendulum motion of the clubhead (kinesthetic)	Swinging motion of the arms(kinesthetic)	
(Lohse et al., 2010)Participants: Novices	Visually focus on the bulls-eye…mentally focus on the movement of your arm. When you're off target think about how you can correct the mistake by changing the motion of your arm. Each time you throw, focus on your arm and think about how you are moving (visual and kinesthetic).	Visually focus on the bulls-eye…mentally focus on the flight of the dart. When you're off target think about how you can correct the mistake by changing the flight of the dart. Each time you throw, focus on your dart and think about how it should fly (visual and kinesthetic).	
Schorer et al. (2012)Participants: Experts and novices	Concentrate on the bullseye (visual)	Internal 1: concentrate on the return point of the dart (kinesthetic) Internal 2: concentrate on the release of the dart (kinesthetic)	

*An exploratory representation of varying focus of attention instructions used in different studies within the context of aiming tasks*.

When focusing on understanding peak performance in the context of perception-action coupling, particularly in aiming sports, the quiet eye phenomenon has received considerable attention in the context of sport science. This perceptual criterion, which focuses on visual information processing, is also seen as a strong predictor of motor outcomes (Vickers, 2007; Rienhoff et al., 2016). The quiet eye reflects the final fixation on a target prior to the initiation of the critical movement phase and has been examined in the context of tactical tasks, interceptive timing tasks, and targeting tasks (Vickers, 2007). The quiet eye has a minimal duration of 100 ms and a maximum gaze vector deviation of 3° (Vickers, 2009). Previous research has linked expertise differences to quiet eye duration with experts showing longer durations (Vickers, 1996). Furthermore, studies have shown that longer quiet eye durations are associated with better motor results (Moore et al., 2012; Vine et al., 2014), especially in aiming tasks (Vickers et al., 2000; Harle and Vickers, 2001).

According to Lebeau et al. (2016), only nine investigations have focused on the influence of quiet eye duration in the context of perceptual-motor learning. In these studies, different instructions and feedback were used to influence perceptual performance (i.e., quiet eye) (Adolphe et al., 1997; Wilson et al., 2009). One of the first studies to investigate the trainability of quiet eye duration was done by Adolphe et al. (1997). They examined changes in quiet eye duration over a 6-week training intervention in volleyball players. Players were given video feedback about their gaze behavior that led to longer durations and earlier onsets of the final fixation. In a subsequent study, Harle and Vickers (2001) considered the influence of a quiet eye training intervention using video feedback over a two season period in basketball players compared with elite models. This training resulted in the basketball players improving their throwing average and demonstrating longer and more stable quiet eye durations. Learning studies have also investigated quiet eye in the context of anxiety, with results showing a positive influence of learning on quiet eye duration (Vine and Wilson, 2011; Wood and Wilson, 2011, 2012). Wilson et al. (2009) investigated basketball free throws under three different conditions (control condition, high, and low pressure) and showed that quiet eye durations were longer for hits compared to misses. Moreover, they showed that quiet eye durations decreased in high pressure situations, whereas the number of fixations increased.

Combining these two phenomena, recent studies have begun explore the influence of focus of attention instructions on quiet eye duration. The existing examinations in this area (Klostermann et al., 2014; Ziv and Lidor, 2014; Rienhoff et al., 2015) have shown mixed results, again perhaps due to the use of varying instructions (see Table 2). Ziv and Lidor (2014) asked participants to perform golf putts after receiving internal and external focus of attention instructions. Additionally, they had participants perform the task under distracted and non-distracted conditions, with results showing a benefit for external instructions on quiet eye duration but not putting performance in non-distracted situations. Rienhoff et al. (2015) investigated differences between external and internal foci of attention, which were spatially relatively close together (external: hand, internal: hand). The results indicated internal focus was beneficial for extending quiet eye durations, but the external focus resulted in better throwing accuracy. Querfurth et al. (2016) showed a benefit for internal instructions in novices by investigating the QE duration and the motor outcome in a dart throwing task resulting in earlier QE-onset and later QE-offset in internal instructions. Last, Klostermann et al. (2014) investigated the influence of movement-related and effect-related focus of attention and demonstrated better putting performance for effect-related instructions and a later quiet eye offset with movement-related quiet eye duration. As noted in Table 2, there were considerable differences in the instructions used across these studies. When considering the instructions in recent studies concerning quiet eye training, it is notable that all instructions focused on kinesthetic parameters, compared with studies on focus of attention (see Table 1), where instructions in both visual and kinesthetic categories were used.

**Table 2:** Varying used focus of attention instructions in the context of quiet eye.

**Study**	**Instruction 1**	**Instruction 2**
Klostermann et al. (2014)	Effect-related: Hit the target cross as accurately as possible and, in particular, mentally pay attention to the feeling when the ball leaves the head of the putter. By this, I mean the first feedback on putting success (feeling virtually no collision between the ball and putter head) or failure (feeling a noticeable collision between the ball and putter head) (kinesthetic).	Movement-related: Hit the target cross as accurately as possible and, in particular, mentally pay attention to the feeling at the rear reversal point of the swing. By this, I mean the rhythm and speed of the swing between backswing and forward swing (kinesthetic).
Rienhoff et al. (2015)	Exernal: focus on the ball (kinesthetic)	Internal: focus on the hand (kinesthetic)
Ziv and Lidor (2014)	External: focus on the pendulum motion of the club head (kinesthetic)	Internal: focus on the swinging motion of their arms (kinesthetic)

*Recently used focus of attention instructions in studies within the context of quiet eye duration and motor performance*.

Moore et al. (2012) considered the influence of a training intervention on quiet eye duration and putting performance in golf novices and used focus of attention instructions as an explanation for their results. Two different groups took part in the study (quiet-eye training group, technical training group) with the quiet eye training group receiving instructions on the direction of their gaze behavior, while the technical training group's instructions related to the technical execution of a golf putt. All participants had to perform baseline, retention and “pressure” tests. In addition to results on physiological parameters, results showed that the quiet eye trained group had longer quiet eye durations and a more expert-like putting performance (kinematic) in retention and pressure tests. Moore et al. (2012) argued that their results may be associated with a better external focus of attention and that longer quiet eye durations lead to a more effective external focus of attention.

Studies to date demonstrate interactions between perceptual phenomena (quiet eye) and cognitive processes (focus of attention) (Klostermann et al., 2014; Ziv and Lidor, 2014; Rienhoff et al., 2015). Rienhoff et al. (2015) postulated that this research area needs greater attention to gain deeper insight into the processes of perception-action coupling, especially regarding the mechanisms that explain the influence of different instructions on the quiet eye and motor performance. Unfortunately, the instructions used across studies have been inconsistent, making it difficult to compare results. As Wulf (2013) postulated, a change of a single word might influence the performance outcome.

The first aim of this study was to replicate findings concerning the trainability of quiet eye duration (Adolphe et al., 1997; Harle and Vickers, 2001; Causer et al., 2011; Vine et al., 2011, 2014) and the association of this duration with motor performance in darts (Vickers et al., 2000). We hypothesized a general improvement in quiet eye duration and throwing accuracy from pre- to post-test. More precisely, we assumed that practice would increase the outcome which is related to participant's performance in both motor and visual behavior using a training intervention with attentional instructions.

Our second aim was to investigate the association between quiet eye duration and focus of attention by classifying focus of attention instructions to allow a better comparison between different studies. To this end, we developed a category system that is more sensitive in order to gain more precision into the association between the phenomena and their influence on the motor result. The similarities and differences in the instructions used in prior work informed the development of two different categories, locus-specific instructions [external vs. internal, such as used by Wulf (2013)], and perceptionspecific instructions [kinesthetic vs. visual such as the movement-related or vision-related foci as used by Lohse et al. (2010), Schorer et al. (2012), Klostermann et al. (2014)]. In the first category, all instructions relate to vision, directing the visual system either directly or indirectly. Conversely, kinesthetic instructions focus on the movement itself (e.g., movement execution, movement of a subject like a ball). The foundation for this approach was the assumption that visual instructions might influence perceptual performance while kinesthetic instructions might influence motor performance, detached from the locus of attention (external vs. internal). Therefore, we investigated the influence of four different focus of attention instructions (internal vs. external x visual vs. kinesthetic) on quiet eye duration and motor performance in the context of visuo-motor learning. Keeping in mind that a single word might change the performance outcome, we tried to use instructions that were very similar. On the one hand, referring to Wulf (2013) we focused on the difference between external and internal focus of attention, while on the other hand we tried to generate two new categories (visual and kinesthetic) which consider the possibility of perception-specific focus of attention. To gain deeper insight, we categorized the examples in Tables 1, 2 to the visual and kinesthetic approach used in this study. In addition, no studies have focused on the association of focus of attention and quiet eye using a focus of attention based on the visual category.

For throwing performance, we hypothesized better throwing results for external instructions compared with internal instructions (locus of attention) based on Wulf (2013). Second, we assumed a higher improvement in throwing accuracy via kinesthetic instructions because of the possible link between motor performance and the kinesthetic sense. As noted, instructions given in previous studies differ a lot from each other, Schorer et al. (2012) focused on kinesthetic instructions (movement), whereas Lohse et al. (2010) used a visual instruction on the bullseye (cf. Table 1). Finally, we hypothesized a benefit (throwing performance) in perception-directed instructions compared to locus-directed instructions.

For quiet eye duration, we hypothesized longer quiet eye durations for the external instructed compared with the internal instructed group (locus-directed focus of attention), based on the assumption that external instructions lead to superior results (Wulf, 2013; Ziv and Lidor, 2014). In addition, we hypothesized a benefit for visually-directed instructions compared with kinesthetic instructions because the perceptual phenomenon of quiet eye might be more affected by a focus of attention related to directing the visual sense. Finally, we hypothesized an improvement (longer quiet eye duration) in perception-directed instructions compared to locus-directed instructions. These instructions might influence visual perception (quiet eye duration) more than locus of instruction, with the greatest benefit expected for external visual instructions. As mentioned, recently used instructions mixed locus-specific (internal vs. external) and perception-specific (visual vs., kinesthetic) instructions, without differentiating between these categories. This differentiation might be fruitful for perceptual-motor learning.

In summary, our differentiation of instructions should facilitate a more detailed investigation of the interaction of locus-specific and perception-specific focus of attention. This might be a first step toward better comparability between studies using different instructions.

## Methods

### Participants

A total of 36 dart novices completed this study and were divided into four groups [internal visual (*n* = 10), external visual (*n* = 9), internal kinesthetic (*n* = 7) and external kinesthetic (*n* = 10)]^1^. Participants had no experience in dart training and had normal or corrected-to-normal vision. At the beginning of this study all participants provided informed consent and completed a questionnaire detailing their age, dart experience and any eye or visual diseases. This study was approved by the University of Oldenburg ethics committee.

### Task and Procedure

The main task in the experiment was to perform dart throws toward the bullseye as accurately as possible. The dartboard was in line with the standards of the World Darts Federation (WDF). The dartboard had a total diameter of 34 cm and the bullseye was adjusted to a height of 1.73 m with a throwing distance of 2.37 m. All participants used regular darts with a weight of 24 g per arrow. The study design was divided into three phases, pretest, training and posttest. First, participants were asked to perform 30 dart throws, aiming to hit the bullseye or get as close as possible. After the pretest, participants were divided randomly into four different groups for the training phase. Participants conducted three training days with 50 throws in each session. Each group was instructed differently in the training phase:

- visual internal (*concentrate on your eye*)
- visual external (*concentrate on the bullseye*)
- kinesthetic internal (*concentrate on your hand*)
- kinesthetic external (*concentrate on the dart*)

The instructions were given exactly as reported above. The word “concentrate” was used to direct each participant's focus of attention. When considering the attentional focus “performer's focus of attention or concentration during the planning or execution of a motor skill has a significant influence on movement quality.” (Wulf, 2013) The whole procedure, including the training interval (pretest, training, posttest), was done in ~7 days per participant.

### Apparatus and Measurement

In the pre- and post-test, quiet eye duration (in ms) and throwing accuracy as radial distance from the bullseye (in cm) were analyzed as dependent variables. For measuring throwing accuracy, an external digital camera (Sony, HDR-CX320, 8.9 megapixels) was used to capture videos from the dartboard. From the recorded videos, screenshots were produced to allow for the analysis of the radial distance from the bullseye in pixels, which were converted into cm. Gaze behavior was recorded using SMI eye tracking glasses 2.0. This head mounted eye tracking system enables binocular eye tracking with a frequency of 60 Hz and facilitates mobile eye tracking by a linked smartphone via USB stored in a belt bag. Thus, participants were able to move freely and carry out dart throws in an almost natural environment. After the eye tracking system was adjusted, a three-point calibration was done to ensure an optimal tracking ratio for each participant. For defining the quiet eye period, the integrated scene camera from the eye tracking glasses was used. This camera recorded the field of vision from the participant. Based on these videos, quiet eye duration was analyzed with BeGaze, a gaze analysis software. In doing so, the returning point (flexion to extension) of the throwing motion was determined from the scene camera. Then, the quiet eye period was extracted by analyzing the last fixation that began prior to the returning point. The duration of this fixation was manually extracted and defined as the quiet eye duration. Therefore, BeGaze also considered the duration the duration as the gaze vector deviation in the automatic analysis as requested by the definition of quiet eye. Random frame-by-frame video analysis was done to check data accuracy. The quiet eye period was defined as being prior to the critical movement phase. In the targeting task of dart throwing the critical movement phase is the start of the extension phase (Vickers et al., 2000).

### Statistical Analyses

To check for baseline differences, an analysis of variance was performed for quiet eye duration and throwing accuracy. If pretest differences were found, an ANCOVA (2x2x2: test × locus-directed focus vs. perception-directed focus) was calculated. If no baseline differences were measured, an ANOVA (2x2x2: test × locus-directed focus vs. perception-directed focus) was conducted. In addition, a Kolmogorov Smirnov Test was calculated to check the normality of the data.The alpha level was set to 0.05 and all data analyses were conducted with SPSS 22.0, Effect size generator 2.3 (Devilly, 2004) and G^*^power 3.1.9.2 (Faul et al., 2007).

## Results

In the following section, the influence of different instructions on perceptual-motor results are described, beginning with the influence on the perceptual performances (e.g., quiet eye) followed by the results for throwing accuracy.

### Results Concerning Quiet Eye Duration

First, we investigated differences in quiet eye duration with a baseline check. No significant differences between locus specific focus of attention, *F*_(1,32)_ = 0.07, *p* = 0.79, *f* = 0.04, *CI* = − 0.52 to 0.79, 1 − β = 0.95, and perception specific focus of attention, *F*_(1,32)_ = 1.37, *p* = 0.25, *f* = 0.40, *CI* = − 0.26 to 1.06, 1 − β = 0.96, were demonstrated. Additionally, no significant interaction was revealed, *F*_(1,32)_ = 0.07, *p* = 0.79, *f* = 0.04, 1 − β = 0.95 (cf. Table 3).

**Table 3:** Mean scores (M) and standard deviation (SD) for the different groups in throwing accuracy (TA) and quiet eye duration (QED).

**Instruction**	**TA Pretest**		**TA Posttest**		**QED Pretest**		**QED Posttest**	
	***M***	***SD***	***M***	***SD***	***M***	***SD***	***M***	***SD***
Internal-visual	6.8	1.2	6.1	1.2	498	322	867	392
External-visual	6.6	1.8	6.7	2	499	347	946	442
Internal-kinesthetic	11	4.4	11.3	2.8	618	380	579	293
External-kinesthetic	10.8	2.9	9.6	3.6	686	482	565	384

**Figure 1:**
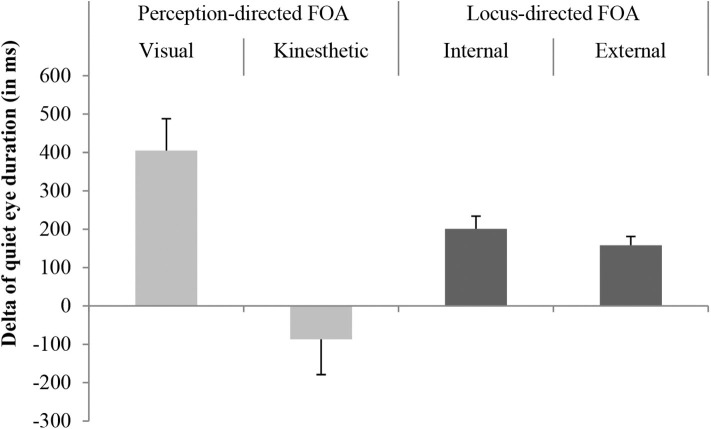
Mean values of the variation of the quiet eye duration between pre- and posttest (Δ) with error bars representing the standard deviation.

To investigate differences in quiet eye duration between tests, we conducted an analysis of variance (2x2x2) (tests × locus-directed focus vs. perception-directed focus), which revealed significant differences between pre- and post-tests, *F*_(1,32)_ = 6.93, *p* = 0.01, *f* = 0.43, *CI*: −0.03 to 0.90, showing a strong effect size. Quiet eye duration was extended from pretest (*M:* 574 ms, *SD*: 382 ms) to posttest (*M:* 747 ms, *SD*: 408 ms), the Δ of the alteration of the quiet eye duration is presented in Figure 1. No significant differences for perception-specific focus of attention were found, *F*_(1,32)_ = 0.62, *p* = 0.44, *f* = 0.24, *CI*: −0.42 to 0.90, but there was a significant interaction of test and perception-specific focus of attention, *F*_(1,32)_ = 15.31, *p* < 0.01, *f* = 0.69. Visually instructed groups increased their quiet eye duration while the kinesthetically instructed group reduced their quiet eye duration. For locus-specific focus of attention no significant differences were revealed, *F*_(1,32)_ = 0.09, *p* = 0.77, *f* = 0.08, *CI*: −0.57 to 0.74, 1 − β = 0.95. As well, no significant interaction of test and locus-specific focus of attention, *F*_(1,32)_ < 0.01, *p* = 0.97, *f* = 0.05, 1 − β = 0.95 was found. Both the externally instructed groups and the internally instructed groups increased their quiet eye duration between tests (c.f. Figure 1). The three way interaction (test × visual vs. kinesthetic × internal vs. external) was also not significant, *F*_(1,32)_ = 0.43, *p* = 0.52, *f* = 0.11, 1 − β = 0.10.

### Results Concerning Throwing Accuracy

To test for baseline differences, we conducted an analysis of variance (2x2x2) (test × internal vs. external × visual vs. kinesthetic). Pretest differences between the two perception specific foci of attention, *F*_(1,32)_ = 20.75, *p* < 0.01, *f* = 1.47, *CI* = 0.74–2.20, were revealed. The visually instructed group demonstrated better throwing accuracy compared to the kinesthetically instructed group. No significant differences were found for the locus specific focus of attention, *F*_(1,32)_ = 0.05, *p* = 0.83, *f* = 0.03, *CI* = − 0.65 to 0.56, 1 − β = 0.95. Additionally, no significant interaction was found, *F*_(1,32)_ < 0.01, *p* = 0.99, *f* < 0.01, 1 − β = 0.95.

**Figure 2:**
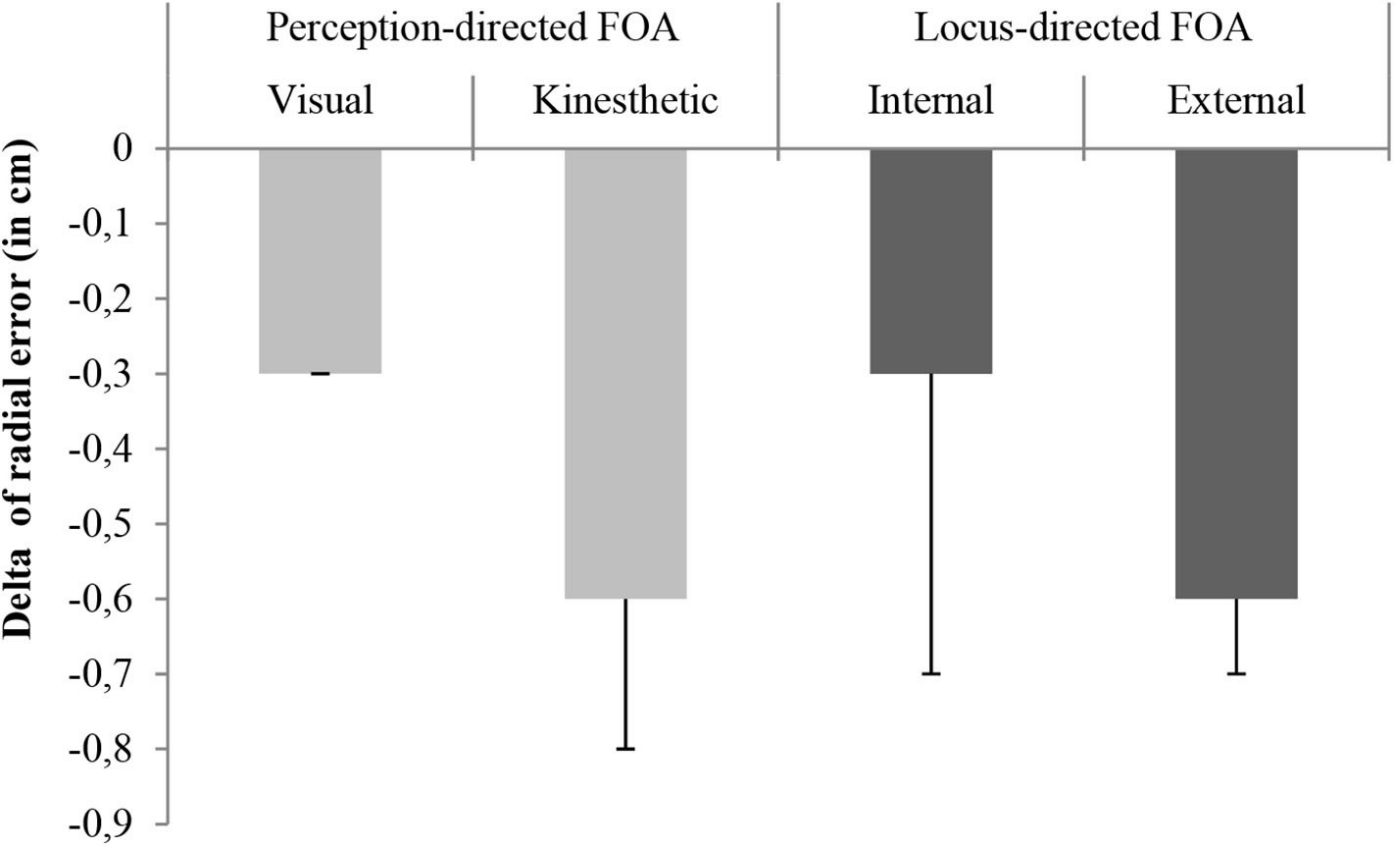
Mean values representing the reduction of radial error in throwing accuracy between pre- and posttest (Δ) with error bars representing the standard deviation.

Due to the pretest differences in the perception specific focus of attention, an analysis of covariance (2x2: internal/external vs. visual/kinesthetic) was done for group differences. The ANCOVA showed no significant interaction for the groups, *F*_(1,31)_ = 3.35, *p* = 0.08, *f* = 0.33, 1 − β = 0.97. Also, no significant differences between the locus-foci of attention *F*_(1,31)_ = 0.40, *p* = 0.53, *f* = 0.11, 1 − β = 0.95, or the perception-specific focus of attention, *F*_(1,31)_ = 2.76, *p* = 0.11, *f* = 0.30, 1 − β = 0.96, were found. To investigate differences for throwing accuracy from pre-to post-test, an ANOVA was conducted but showed no significant effects, *F*_(1,32)_ = 1.13, *p* = 0.30, *f* = 0.13, *CI* = − 0.33 to 0.59, 1 − β = 0.95. All groups improved throwing accuracy slightly with lower values representing a better accuracy (Pretest: *M*, 8.7 cm; *SD*, 3.3 cm/ Posttest: *M*, 8.2 cm; *SD*, 3.2 cm). The Δ of the radial error reduction is presented in Figure 2.

## Discussion

The first aim of this investigation was to replicate findings concerning the trainability of quiet eye duration and its association with motor results. As expected, we were able to replicate findings regarding the trainability of quiet eye; the 1-week training intervention led to significantly longer quiet eye durations overall. However, this effect was different across the training groups. Quiet eye durations increased in the visually instructed groups, but the kinesthetically instructed groups showed reduced quiet eye durations.

Interestingly, there were descriptive results showing that the groups improved their throwing results by reducing radial error, and although these results did not reach significance they showed a reasonable effect size (*f* = 0.13) given the length of the intervention. The non-significant improvement of throwing accuracy from pre to post-test might be best explained with the measuring unit. When having a look at the dartboard, millimeters are crucial for a hit or a miss. It might be that a longer training intervention would lead to greater reductions in the radial error but we believe this positive trend is notable. Pre-/post-test differences showed longer quiet eye durations with a reduction in radial error and therefore better motor results. These results are in line with prior work (Harle and Vickers, 2001; Vine and Wilson, 2010; Vine et al., 2013; Miles et al., 2014). In summary, these trends suggest an improvement in quiet eye duration is associated with better throwing results.

Our second aim of this study was to evaluate the different focus of attention instructions, particularly the value of more sensitive instructions for gaining deeper insight into the association between quiet eye duration and throwing results in darts. When considering the locus of focus of attention (external vs. internal, Hypothesis 1), no significant group differences were revealed for throwing accuracy but there was a medium sized effect (*f* = 0.33). It is notable that the trend suggests a higher improvement for the external compared to the internal instructed groups, which is descriptively in line to our hypothesis and prior work (Carpenter et al., 2013; Wulf, 2013). When considering the results on quiet eye duration, no significant group differences were found. Both locus-directed groups increased their quiet eye duration.

Our second hypothesis considered differences in the perception-specific focus of attention (visual vs. kinesthetic). No significant group differences were found for throwing accuracy. However, besides the externally instructed groups, the kinesthetically instructed groups showed a lot higher improvement in throwing accuracy compared with visual or internal instructions. For quiet eye duration, a significant interaction between perception-specific groups and test was revealed. The visually instructed group increased their quiet eye duration whereas the kinesthetically instructed group decreased their quiet eye duration. This is in line with our hypothesis that in addition to locus of instruction, the sense that is being targeted in the instruction can influence the perceptual and motor result.

For the comparison between locus- and perception-specific focus of attention, no significant interaction of groups was revealed for throwing accuracy: all groups improved throwing accuracy slightly. Similarly, the interaction for quiet eye duration was also not significant. Surprisingly, we were not able to replicate the significant group differences between the locus-directed instructions (internal vs. external) in novices; whenever the trend showed the correct direction in throwing accuracy, the results for quiet eye duration were very similar. These results are in line with prior research (e.g., Wulf, 2013) suggesting novices profit from an external focus of attention.

As Castaneda and Gray (2007) postulated, a focus on skill execution improves motor performance in novices, we would argue that in perceptual-motor learning it is necessary to consider the skill under examination (perceptual or motor), because the specificity of instruction (i.e., kinesthetically vs. visually-directed) may be important. Results concerning our second hypothesis are in line with prior research showing that a focus on the movement effect (external) results in better motor performance then attending to the movement itself (internal) in novices (Wulf and Prinz, 2001). Similarly, Castaneda and Gray (2007) showed that an external focus on skill execution led to better batting performances. With regard to our aims (i.e., benefit for perception-specific focus of attention and its specificity in perceptual-motor learning), one needs to consider that perceptual and motor performance might be differentially influenced by different foci of attention. Concerning the motor results (throwing accuracy), the kinesthetic-external group showed the highest improvement in throwing accuracy, which is in line with results from (Castaneda and Gray, 2007). One possible explanation for this pattern of results is the common-coding theory of perception and action, first discussed in this area by Wulf and Prinz (2001). This theory proposes that actions are controlled by their intended effect. These effects should be as remote as possible and relatively close to the action that produces it. To focus on the dart (external kinesthetic) is less remote as a focus on the hand; moreover, the dart is relatively close to the produced action. Based on this theory, a focus on the dart is directly associated with the movement of the dart throw; however, a focus on the trajectory of the dart, for example, would have led to worse performance because this focus cannot be associated with the movement itself.

Bringing these approaches (Wulf and Prinz, 2001; Castaneda and Gray, 2007) together, it is reasonable that the focus on the dart (kinesthetic external) led to the highest improvement on the motor result in the context of motor learning. When focusing on perceptual performance, one could argue that the visual-external focus of attention should show the highest improvement in quiet eye duration, which is supported by the results of this study. Additionally, the common coding theory of perception and action might explain the pattern for the quiet eye results. Wulf and Prinz (2001) noted that actions might be more fruitful when they are planned with a focus on the intendent effect instead of the movement itself. This approach might explain the highest benefit of quiet eye duration for visual-external instruction. In particular, the significant interaction of perception-specific focus of attention and test suggests that in addition to locus, the sense being targeted (visual, kinesthetic) influences both motor and perceptual performance. Interestingly it seems that the visual system is affected by both visual foci of attention instructions.

In conclusion, our results indicate that small changes in instructions influence their effects on perceptual and motor results. It is notable that visually-directed instructions showed greater effects on perceptual performance (e.g., quiet eye), and that kinesthetically-directed instructions seemed to influence the motor result (radial error) as strongly as the external instructions. Results of this 1-week training intervention suggest perceptual and motor learning in novices could be asynchronous. With this in mind, it would be interesting to have a closer look at the synchrony of perceptual and motor learning; a learning intervention of 1 week might be not long enough to show the assimilation of these two factors. Further research should focus on longer training interventions for getting deeper insight into the influence of instructions in the learning of perceptual-motor skills and on perception and action coupling in motor learning processes. Ziv and Lidor (2014) argued that the influence of different focus of attention instructions might be task specific and dependent on the skill level of the learner. Moreover, they argued that more work on specific foci is necessary for getting a deeper understanding of the phenomenon, especially with different levels of expertise and specific tasks. Given our results, the necessity of a standardized and well categorized instruction system is obvious; unfortunately this has not been done in prior work. So far, many studies have used inconsistent instructions making it difficult to compare across studies. This study is a first step to categorizing instructions (possibly only relevant for aiming tasks) that developed two more sensitive categories (locus specific: external vs. internal, perception-specific: visual vs. kinesthetic), which seem to be useful in the context of perceptual-motor learning.

Despite these interesting results, it is possible there were limitations in the methodological design (e.g., difficulty for the participants to interpret the instruction *focus on your eye and the sample size*) suggesting our approach needs to be validated in further research. Additionally, the low sampling rate of the eye tracker (60 Hz) may have influenced the results, higher sampling rates might show a better resolution of fixation duration and should be used in next studies. It is also possible our category system does not consider all categories; as noted earlier, the influence of focus of attention instructions seems to be task specific and there might be additional categories, as previous research pointed out (Hänsel and Seelig, 2003; Hossner et al., 2006). However, the categories used in this study provide a good basis to develop a system that promotes better comparability between the instructions used across studies. Our study showed that besides the locus of attentional focus, the perception-directed focus seems to influence perceptual-motor learning in novices. It will be important to determine the extent to which the effect of perception-directed focus of attention applies beyond aiming tasks. While our study provides new insight on the association of focus of attention and the quiet eye, additional work will be necessary to gain further understanding of other perceptual-motor tasks, different expertise levels and the associations between these factors with perceptual, cognitive and motor performance.

## Data Availability Statement

The datasets generated for this study are available on request to the corresponding author.

## Ethics Statement

The studies involving human participants were reviewed and approved by Ethical committee of the University of Oldenburg. The patients/participants provided their written informed consent to participate in this study.

## Author Contributions

JN and JS planned and conducted the study. All authors contributed to the article and approved the submitted version.

## Conflict of Interest

The authors declare that the research was conducted in the absence of any commercial or financial relationships that could be construed as a potential conflict of interest.

## Footnotes

^1^The differences in the sample sizes for each group were due to four participants dropping out during the training phase of this study.
